# Exploring the Potential of *Bacillus subtilis* IS1 and *B. amyloliquificiens* IS6 to Manage Salinity Stress and *Fusarium* Wilt Disease in Tomato Plants by Induced Physiological Responses

**DOI:** 10.3390/microorganisms12102092

**Published:** 2024-10-19

**Authors:** Waheed Akram, Shama Sharif, Areeba Rehman, Tehmina Anjum, Basharat Ali, Zill-e-Huma Aftab, Ayesha Shafqat, Laiba Afzal, Bareera Munir, Humaira Rizwana, Guihua Li

**Affiliations:** 1 Department of Plant Pathology, Faculty of Agricultural Sciences, University of the Punjab, Lahore 54590, Pakistanshamasharif1798@gmail.com (S.S.); tehminaanjum@yahoo.com (T.A.); 2 Vegetable Research Institute, Guangdong Academy of Agricultural Sciences, Guangzhou 510640, China; areebarehman453@gmail.com; 3 College of Earth and Environmental Sciences, University of the Punjab, Lahore 54590, Pakistan; laibaafzal2233@gmail.com (L.A.); bareeramunir@yahoo.com (B.M.); 4 Institute of Microbiology and Molecular Genetics, University of the Punjab, Lahore 54590, Pakistan; basharat.ali.mmg@pu.edu.pk; 5 School of Botany, Minhaj University, Lahore 54770, Pakistan; ayesha.bot@mul.edu.pk; 6 Department of Botany and Microbiology, College of Science, King Saud University, Riyadh 11495, Saudi Arabia; hrizwana@ksu.edu.sa

**Keywords:** salt tolerance, *Fusarium* wilt, *B. subtilis*, *B. amyloliquificiens*, rhizobacteria, climate change

## Abstract

The intensified concerns related to agrochemicals’ ecological and health risks have encouraged the exploration of microbial agents as eco-friendly alternatives. Some members of *Bacillus* spp. are potential plant-growth-promoting agents and benefit numerous crop plants globally. This study aimed to explore the beneficial effects of two *Bacillus* strains (*B. subtilis* strain IS1 and *B. amyloliquificiens* strain IS6) capable of alleviating the growth of tomato plants against salinity stress and *Fusarium* wilt disease. These strains were able to significantly promote the growth of tomato plants and biomass accumulation in pot trials in the absence of any stress. Under salinity stress conditions (150 mM NaCl), *B. subtilis* strain IS1 demonstrated superior performance and significantly increased shoot length (45.74%), root length (101.39%), fresh biomass (62.17%), and dry biomass (49.69%) contents compared to control plants. Similarly, *B. subtilis* strain IS1 (63.7%) and *B. amyloliquificiens* strain IS6 (32.1%) effectively suppressed *Fusarium* wilt disease and significantly increased plant growth indices compared to the pathogen control. Furthermore, these strains increased the production of chlorophyll, carotenoid, and total phenolic contents. They significantly affected the activities of enzymes involved in antioxidant machinery and the phenylpropanoid pathway. Hence, this study effectively demonstrates that these *Bacillus* strains can effectively alleviate the growth of tomato plants under multiple stress conditions and can be used to develop bio-based formulations for use in the fields.

## 1. Introduction

In the past few years, there has been a worldwide increase in soil salinization, leading to challenges to food security in numerous countries [1]. Salinity stress is widespread and severely hinders the yield of crops both in arid and semi-arid regions [2]. According to an estimate, >30% of irrigated areas are salinity-affected to varied extents, which will spread up to 50% by 2050 [3]. Salinity in soil adversely affects various aspects of plant characteristics, including chemical, physical, and biological attributes. Additionally, it hampers processes such as photo-assimilation, the uptake of essential nutrients, transpiration, water release protein, production, and the control of plant hormones, leading to diminished plant development [3].

*Solanum lycopersicum* (tomato) belongs to the Solanaceae family. Certain varieties of tomatoes are moderately salt-tolerant [4,5]. In general, *S. lycopersicum* can tolerate salinity levels up to 3 dS m^−1^ without causing any apparent damage [6]. However, being a sensitive halotolerant species, at high salinity levels, tomato plant growth can significantly decline and affect the plant’s overall productivity [7].

Besides the abiotic factors, various biotic stresses, including plant diseases, have also limited crop productivity in tomato plants [8]. Biotic stress such as the *Fusarium* wilt disease triggered by a soil-borne fungus, *Fusarium oxysporum* f.sp. *lycopersici*, is considered the most destructive and economically damaging disease of tomatoes [9]. The characteristic symptoms include wilting, chlorosis, stunted seedlings, and reduced leaf size [9].

It was reported in many studies that plant-growth-promoting rhizobacteria (PGPR), in both abiotic and biotic stresses, can enhance and improve the overall growth of various crops [10,11]. PGPRs have been shown to not only improve various growth parameters under saline stress conditions, including increased stem elevation, root length, and both dry and fresh weight, but they can also produce various hormones like indole-3-acetic acid and 5-aminolevulinic acid [12,13]. Additionally, PGPRs also aid in nitrogen, potassium, phosphate, and solubilized uptake, as well as siderophore production, by triggering the systemic defense responses in cucumber seedlings under saline conditions [14]. *Bacillus* are Gram-positive, ubiquitous, aerobic bacteria that are considered active members of soil microbiota and found near plant roots [15]. *Bacillus*–plant interactions are diverse and governed by a wide array of metabolites to enhance plant growth and defenses against invading pathogens [16]. *Bacillus* makes them thrive in unconducive environments [17]. After germination, chemotaxis steers these bacteria towards plant root colonization and benefits the host plant [18]. After successful colonization, these beneficial microbes elicit growth promotion and enhance the defense state under various stress conditions [19]. Different strains of *Bacillus* are biocontrol agents against plant pathogens and can also mitigate abiotic stress. The responses to manage biotic and abiotic stresses are due to the ability to produce lytic enzymes, antibiotic substances, and metabolic regulations in plants to restore the distorted production of primary and secondary metabolites inside the plant body [20,21,22].

In our recent study, we reported two *Bacillus* strains (*B. subtilis* strain IS1 and *B. amyloliquificiens* strain IS6) with antagonistic activity, which led to the protection of pea plants against *Fusarium* wilt disease and plant growth promotion [23]. Hence, it seems probable that these beneficial *Bacillus* microbes can ameliorate tomato growth under biotic and abiotic stress. Thus, the objectives of the current investigation were as follows: (1) to examine the beneficial effect of both *Bacillus* strains provided via soil drenching on the suppression of *Fusarium* wilt disease; (2) to improve tomato plant growth under salinity stress; (3) to elucidate the mechanisms behind stress amelioration in tomato plants by both *Bacillus* strains; and (4) to investigate the correlation among different examined parameters for both biotic and abiotic stress amelioration separately.

## 2. Materials and Methods

### 2.1. Microbial and Plant Resources

Seeds of the tomato variety Roma (Lot No.: ZE1448B; Ch. Ahmad Din and Sons, Gujranwala, Pakistan) were purchased from the local market. Two test bacteria strains, i.e., *B. subtilis* strain IS1 and *B. amyloliquificiens* strain IS6, were retrieved from our in-house culture collection and maintained on a Luria–Bertani Agar (LBA) medium inside culture tubes. The virulent isolate of *Fusarium oxysporum* f.sp. *lycopersici* was cultured on a potato dextrose agar (PDA) medium. This pathogen isolate was isolated from infected tomato plants obtained from the experimental station of the Department of Plant Pathology, University of the Punjab, Lahore, Pakistan, and has been used in our previous studies [24]. 

### 2.2. Analysis of the Plant Growth Promotion

Tomato seeds were surface sterilized by submerging into 70% ethanol for 30 s and 1% sodium hypochlorite for another 60 s. Afterward, seeds were washed with distilled sterilized water several times and sown in 12-inch-diameter pots containing 4.5 kg of autoclaved soil randomly collected from a depth of 0 to 10 cm from the experimental station of the Department of Plant Pathology, University of the Punjab, Lahore, Pakistan. The physiochemical properties of soil are provided in Supplementary Table S1. The pots were kept in the greenhouse with temperatures ranging from 14 to 30 °C under a natural photoperiod with 9 to 12 light hours. The plants were irrigated using distilled autoclaved water in 4- to 6-day intervals. The plants were inoculated with bacterial strains after 20 days of emergence. For that purpose, bacterial strains were multiplied separately in the liquid Luria–Bertani broth medium overnight, and cells were collected after centrifugation in sterilized autoclaved water. The optical density (OD) was adjusted to 0.5 at 600 nm. A total of 50 mL of the aqueous formulation of both bacterial strains was added in the region near plant roots after the gentle scraping of the soil. The control plants received only sterilized water. The experiment was laid in a randomized block design (RBD). After six weeks of incubation, plants were gently removed and washed in running tap water, and the shoot length and dry biomass were noted. Five biological replicates were included in each treatment and were repeated twice. 

### 2.3. Selection of Salinity Stress Level

Tolerance to saline stress varies significantly across different tomato varieties and/or genotypes. Therefore, an experiment was performed to select suitable salinity levels for tomato plants for further experimentation. Throughout the experiment, pots containing sterilized media were irrigated with saline waters of varying strengths (0, 50, 100, 150, and 200 mM). The salinity solution was provided for up to 75% of the field capacity to ensure uniform saline stress treatment. Tomato plants were raised from sterilized seeds in the salinized pots under the greenhouse conditions mentioned above. Control plants were irrigated with distilled autoclaved water. After 40 days of emergence, shoot length and dry biomass were observed. The experiment consisted of five replicates and was repeated twice. 

### 2.4. Confirmation of Pathogenicity

Another independent pot experiment was conducted to confirm the pathogenicity of *F. oxysporum* f.sp. *lycopersici* on the tomato variety “Roma” being used in this study. *F. oxysporum* was grown on PDA media for two weeks. Conidia were scraped in distilled sterilized water, and their concentration was adjusted to 10^3^–10^4^ conidia mL^−1^ using a hemocytometer. Tomato plants were raised from sterilized seeds and inoculated with the aqueous suspension of *F. oxysporum* after two weeks of emergence. Plants were kept for incubation for another two weeks. During this period, plants were irrigated with distilled autoclaved water. Afterward, disease scoring was performed using a disease assessment scale previously reported [25], and the percent disease index and disease control effect were calculated using the following formula [26].

$$Percent\;Disease\;Index\;\left(\%\right)=\frac{ƩNumber\;of\;diseased\;plants\times Disease\;scale\;value}{Total\;number\;of\;plants\;assessed\times Maximum\;rating}\times 100$$

$$Disease\;Control\;Effect\;\left(\%\right)=\frac{Disease\;index\;of\;control-Disease\;index\;of\;treatment}{Disease\;index\;of\;control}\times 100$$

### 2.5. Analysis of Bacterial Strains in Managing Multiple Stresses in Tomato Plants

The aqueous suspension of PGPR was prepared by inoculating bacterial cells in sterilized LB broth media inside the conical flasks amended with NaCl (150 mM) to ensure their tolerance and survival during pot trials. After inoculation, flasks were kept in a shaking incubator (Wisd, Daihan, Seoul, Republic of Korea) for 24 h at 25 °C and 100 rpm. The next day, cells were separated by centrifugation at 8870× *g* for twenty minutes. Bacterial cells were resuspended in distilled autoclaved water, and OD was adjusted to 0.1 at 600 nm. 

Tomato seedlings were raised in 4-inch-diameter plastic pots filled with the sterilized silt loam soil as a potting mix, as mentioned in Section 2.2. Pots were irrigated with distilled autoclaved water at intervals of 4 to 6 days. After ten days of emergence, pots were drenched with 50 mL of bacterial inoculum separately for both bacterial strains according to the experimental design (Table 1). The control group was provided with an equal amount of distilled autoclaved water. After three days of bacterial application, plants were challenged with the biotic and abiotic stress agents according to the experimental design (Table 1). Salinity stress was established by irrigating the plants with saline water (NaCl 150 mM) at 75% of the field capacity per pot. On the other hand, biotic stress was applied by pouring 50 mL of the aqueous suspension of *F. oxysporum*, which was prepared as mentioned in Section 2.4. Except for pots under salinity stress (SS) treatments, plants were irrigated with distilled autoclaved water in 4- to 6-day intervals. Plants were incubated for another fifteen days after the biotic and abiotic stress challenge. Ten biological replicates were part of each treatment, and the whole experiment was repeated twice.

### 2.6. Harvest

After twenty days of treatment application, plant growth parameters, e.g., shoot length, root length, fresh biomass, and dry biomass, were recorded. The disease index was calculated from the biotic stress group, as mentioned in Section 2.4. 

### 2.7. Analysis of Change in Chlorophyll and Carotenoid Levels

Leaves were removed from five randomly chosen plants at the final harvest and crushed in liquid nitrogen. One gram of leaf material was extracted with 10 mL of 80% acetone. The extracted material was centrifuged at 8870× *g* for ten minutes. The clear supernatant was used for OD measurement at 663 and 645 nm. The obtained values were added to the equations to obtain quantities of chlorophyll and carotenoid contents [27].

Chlorophyll ‘a’ = (12.21 × OD_663_ − 2.81 × OD_646_)

Chlorophyll ‘b’ = (20.13 × OD_646_ − 5.03 × OD_663_)

Total Chlorophyll = 7.18 × OD_633_ + 17.32 × OD_646_

Carotenoid = [1000 × OD_470_ − 3.27 × {(chl a − 104) × chl. b}]/229

### 2.8. Analysis of the Activities of Total Phenolic Contents and Biotic and Abiotic Stress-Related Enzymes

After one week of treatment application, the quantification of total phenolic contents and enzymes involved in biotic and abiotic stress tolerance was performed according to the experimental design. Total phenolic contents and defense-related enzymes, e.g., peroxidase (POD,) polyphenol oxidase (PPO), and phenylalanine ammonia-lyase (PAL), were quantified from the biotic stress treatments, including UC, Fol, Fol + IS1, and Fol + IS6. On the other hand, the activities of antioxidant machinery enzymes, e.g., peroxidase (POD), catalase (CAT), superoxide dismutase (SOD), and ascorbate peroxidase (APX), were quantified from abiotic stress treatments, including UC, SS, SS + IS1, and SS + IS6. 

For the quantification of total phenolic contents, leaves were removed and crushed into a fine powder using liquid nitrogen. Briefly, 100 mg of leaf material was crushed and extracted in 2 mL of 80% methanol. After centrifugation at 11,320× *g* for 20 min, a clear supernatant was used to quantify total phenolic contents by the Folin–Ciocalteu reagent method [28]. 

For enzyme quantification, one gram of crushed material was extracted in 5 mL of phosphate buffer (100 mM, pH 7). The buffer additionally contained 0.1 g of polyvinylpolypyrrolidone and EDTA at a concentration of 0.1 mM. The mixture was centrifuged at 11,320× *g* for 20 min at 4 °C, and the clear supernatant was used to quantify enzyme activity.

Briefly, peroxidase (POD) was quantified by reacting the enzyme extract with a guaiacol substrate as suggested by Chen et al. [29]. Polyphenol oxidase (PPO) and phenylalanine ammonia-lyase (PAL) were quantified using catechol and L-phenylalanine substrates following the methodologies of Mozzetti et al. [30] and Whetten and Sederoff [31], respectively. The Beauchamp and Fridovich [32] method was used to measure the activity of superoxide dismutase (SOD). Catalase (CAT) and ascorbate peroxidase (APX) enzyme activities were evaluated by observing the breakdown of hydrogen peroxide and ascorbic acid as per the methodologies of Aebi [33,34].

### 2.9. Data Analysis 

One-way analysis of variance (ANOVA) and Tukey’s HSD test (*p* < 0.05) were performed using Excel 2019 software add-in DSAASTAT developed by Onofri (Perugia, Italy). The correlation analysis was performed by using Origin 2018 software (Northampton, MA, USA).

## 3. Results

### 3.1. Effect of Bacillus strains on Plant Growth Promotion

**Figure 1:**
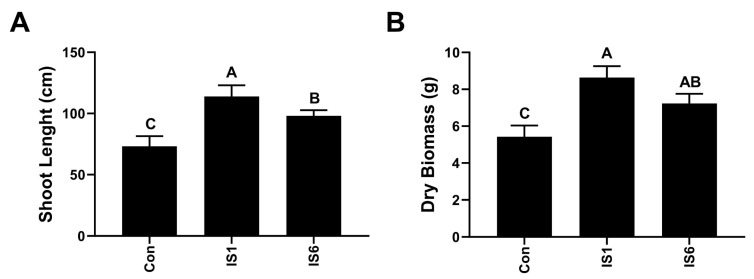
The growth-promoting effect of PGPR on tomato plants. Shoot length (**A**) and dry biomass (**B**) changes under the effect of *B. subtilis* strain IS1, *B. amyloliquificiens* strain IS6, and in the control group. Vertical bars show standard error. Capital letters indicate significant differences among treatments in Tukey’s HSD test (*p* < 0.05).

Two strains were previously isolated from the rhizosphere soil of crop plants in the Punjab province of Pakistan. In this experiment, we analyzed the growth-promoting effect of both bacterial strains on tomato plants during pot trials. The growth indices of tomato plants after PGPR treatment are shown in Figure 1. The shoot length of tomato plants after *B. subtilis* IS1 and *B. amyloliquificiens* IS6 application significantly improved by 57.6% and 32.1% than that in the control, respectively (Figure 1A). Similarly, compared to the control, the plant dry biomass increased by 41.4 and 22.3% for bacterial strains IS1 and IS6, respectively (Figure 1B).

### 3.2. Selection of Salinity Stress Level and Confirmation of F. oxysporum Pathogenicity

**Figure 2:**
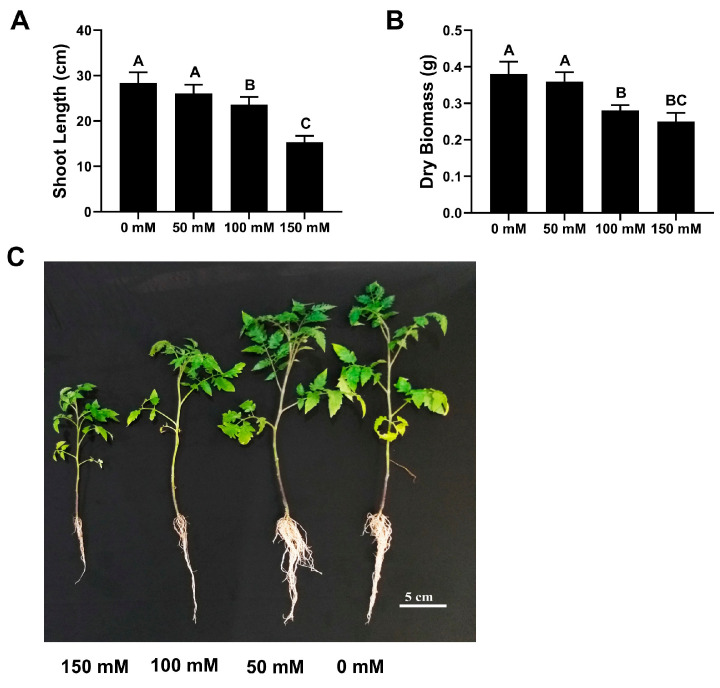
Effect of exposure to different salinity levels on shoot length (**A**) and dry biomass (**B**) accumulation of tomato plants. Vertical bars show standard error. Capital letters indicate significant differences among treatments in Tukey’s HSD test (*p* < 0.05). (**C**) Tomato plants effected by different salinity levels.

**Figure 3:**
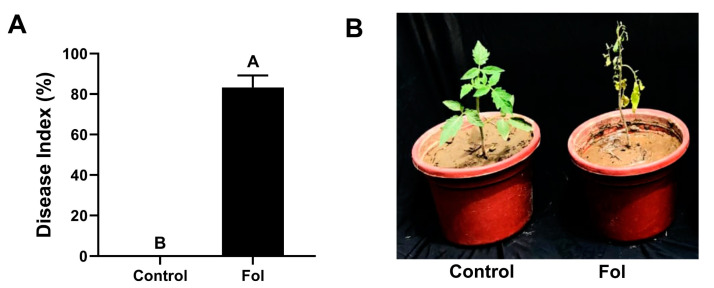
Confirmation of the pathogenicity of *Fusarium oxysporum* f.sp. *lycopersici* (Fol) isolate used in this study. Disease index (**A**) was analyzed two weeks after pathogen application. Vertical bars show standard error. Capital letters indicate significant differences among treatments in Tukey’s HSD test (*p* < 0.05). (**B**) Wilting symptoms on tomato plants.

We conducted preliminary experiments to select suitable salinity levels and confirm *F. oxysporum* pathogenicity for downstream experiments (Figure 2 and Figure 3). Seedlings collapsed right after emergence for 200 mM NaCl application; hence, no growth was possible. For the rest of the salinity levels, the findings showed that 150 mM NaCl caused a maximum reduction in plant height (51.3%) and dry biomass (36.2%) compared to 0 mM (control) treatment (Figure 2). 

Pathogenicity tests performed using a pot trial method with *F. oxysporum* showed the ability of pathogen isolates to cause disease on tomato plants successfully. The plants developed aerial yellowing in the initial symptoms observed during the first week. On the other hand, the whole plant wilted and collapsed in later stages. This experiment proved the virulence of the *F. oxysporum* isolate and the susceptibility of the tomato variety towards *Fusarium* wilt disease used in this study, as shown in Figure 3.

### 3.3. Analysis of Bacterial Strains to Manage Multiple Stress in Tomato Plants

**Figure 4:**
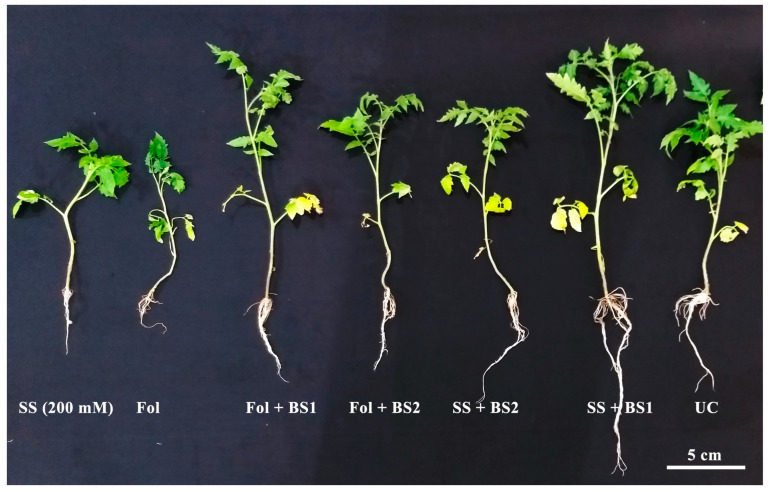
Potential of *B. subtilis* strain IS1 and *B. amyloliquificiens* strain IS6 to suppress salinity stress and *Fusarium* wilt disease in tomato plants. SS = Salinity stress; Fol = *F. oxysporum* f.sp. *lycopersici*; UC = Untreated control.

The ameliorative effects of PGPR treatment with *B. subtilis* (IS1) and *B. amyloliquificiens* (IS6) on tomato plants under salinity and *Fusarium* wilt diseases are displayed in Figure 4. Considering salinity stress, the comparatively best-growth ameliorative effect was obtained by *B. subtilis* (IS1). The results show that the shoot length (45.74%), root length (101.39%), fresh biomass (62.17%), and dry biomass (49.69%) of tomato plants treated by *B. subtilis* (IS1) significantly increased compared to salinized (150 mM NaCl) control plants (Table 2). *B. amyloliquificiens* (IS6) also increased the growth indices of tomato plants under salinity stress. Compared with the salinized control plants, the shoot length and root length increased by 23.92%, and 31.11%, respectively. The fresh and dry weight increased by 26.45% and 23.03% in the same scenario (Table 2 and Figure 4). 

**Figure 5:**
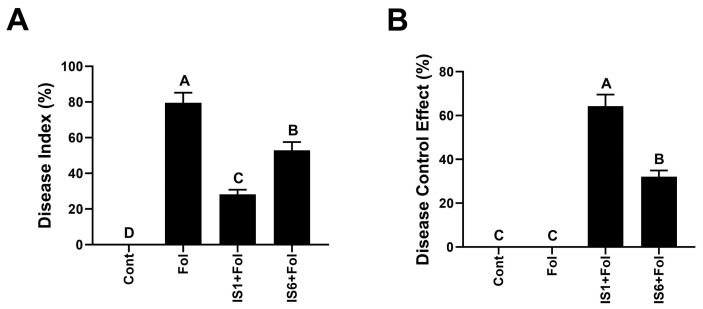
Potential of *B. subtilis* strain IS1 and *B. amyloliquificiens* strain IS6 to suppress *Fusarium* wilt disease of tomato during pot trials. (**A**) = Disease index; (**B**) = Disease control effect; Fol = *F. oxysporum* f.sp. *lycopersici.* Vertical bars show standard error. Capital letters indicate significant differences among treatments in Tukey’s HSD test (*p* < 0.05).

For *Fusarium* wilt disease suppression, both bacterial microbes showed different disease suppression efficiency on tomato plants (Figure 4 and Figure 5 and Table 2). The bacterial strain with the highest disease suppression and plant growth promotion effect was *B. subtilis* (IS1). IS1 suppressed *Fusarium* wilt disease by up to 63.7% compared to the pathogen control treatment (Figure 5). *B. amyloliquificiens* reduced disease by up to 32.06% compared to the pathogen control. Additionally, IS1 significantly increased the shoot length (57.60%), root length (36.53%), and dry weight (63.63%) of tomato plants compared to the disease control (Figure 4 and Table 2). IS6 also increased the growth indices of tomato plants under *Fusarium* stress. Compared with the pathogen control, the shoot length, root length, fresh biomass, and dry biomass were increased by 27.59-, 29.60-, 50.03-, and 54.44%, respectively.

### 3.4. Effect of Bacterial Strains on Chlorophyll and Carotenoid Contents under Stress Conditions

Table 3 shows that the salinity and *Fusarium* wilt disease stress significantly decreased the quantities of chlorophyll contents. Salinity stress decreased chlorophyll a (24.29%), chlorophyll b (30.64%), and total chlorophyll (32.53%) contents compared to non-treated control plants. Here, *B. subtilis* IS1 significantly increased chlorophyll a (39.50%), chlorophyll b (32.33%), and total chlorophyll (37.50%) contents compared to salinity stress control plants (Table 3). Similarly, IS1 increased carotenoid contents up to 23.07% compared to salinity stress control (Table 3). 

Under *Fusarium* wilt stress conditions, chlorophyll a, chlorophyll b, and total chlorophyll contents decreased by 42.05-, 54.68-, and 44.57% compared to non-treated control plants (Table 3). Here, *B. subtilis* IS1 also attenuated the negative effect of *Fusarium* wilt disease on photosynthetic machinery (Table 3). The increase in chlorophyll a, chlorophyll b, and total chlorophyll contents ranged from 54.83-, 27.58-, and 42.39% by *B. subtilis* IS1 application compared to disease control plants (Table 3). Carotenoid contents increased by 19.04% in the same scenario (Table 3). 

Furthermore, the ameliorative activity of *B. amyloliquificiens* IS6 against salinity and *Fusarium* wilt stress was also detected for chlorophyll and carotenoid contents but to a lower extent compared to the *B. subtilis* IS1 (Table 3).

### 3.5. Effect of Bacterial Strains on Abiotic Defense-Related Machinery of Tomato Plants

**Figure 6:**
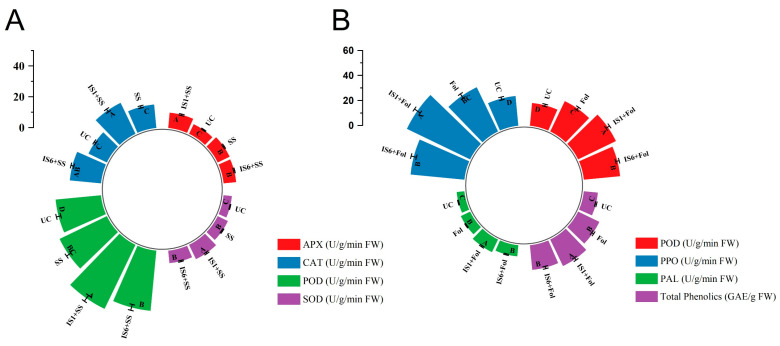
Effect of *B. subtilis* strain IS1 and *B. amyloliquificiens* strain IS6 inoculation on total phenolic, defense, and antioxidant-related enzyme activities of tomato plants under salinity (**A**) and *Fusarium* wilt disease (**B**) stress. Capital letters indicate significant differences among treatments in Tukey’s HSD test (*p* < 0.05). IS1 = *B. subtilis*; IS6 = *B. amyloliquificiens*.

The treatments of *B. subtilis* and *B. amyloliquificiens* resulted in increased activities of antioxidant machinery enzymes (APX, CAT, POD, and SOD) with significant differences from salinity control tomato plants. For both PGPR strains, the activities of all enzymes were higher by 10.8–46.7% compared to tomato plants raised under salinity stress alone (Figure 6A). *B. subtilis* in combination with salinity stress led to 18.4-, 46.7-, 35.7-, and 41.1% increases in APX, CAT, POD, and SOD activities compared to the tomato plants raised under salinity stress alone (Figure 5A). On the other hand, *B. amyloliquificiens* increased CAT, POD, and SOD activities by up to 33.4-, 15.0-, and 10.8%, respectively, in a similar regard (Figure 6A). The onset of salinity stress alone increased activities of APX, CAT, POD, and SOD in tomato plants compared to the non-treated control group but to a lower extent and the application of PGPR further boosted enzyme activities. 

### 3.6. Effect of Bacterial Strains on Biotic Defense-Related Machinery of Tomato Plants

During *Fusarium* stress conditions, a significant increase was observed in the total phenolic content and enzyme activities related to the phenylpropanoid pathway in tomato plants by applying *B. subtilis* and *B. amyloliquefaciens* (Figure 6B). Tomato plants treated with *B. subtilis* in the presence of the *Fusarium* wilt pathogen possessed 24.8% increased total phenolic contents compared to the pathogen control treatment (Figure 6B). Similarly, PO, PPO, and PAL activities increased by up to 43.2-, 35.5-, and 24.9% in *B. subtilis* + *F. oxysporum*-treated plants compared to plants under pathogen-only treatment (Figure 6B). A more pronounced increase in the activities of defense-related compounds was seen under PGPR symbiosis in contrast to the pathogen alone when a comparison was made with the non-treated control plants (Figure 6B).

### 3.7. Correlation Analysis of Plant Physicochemical and Growth Parameters

**Figure 7:**
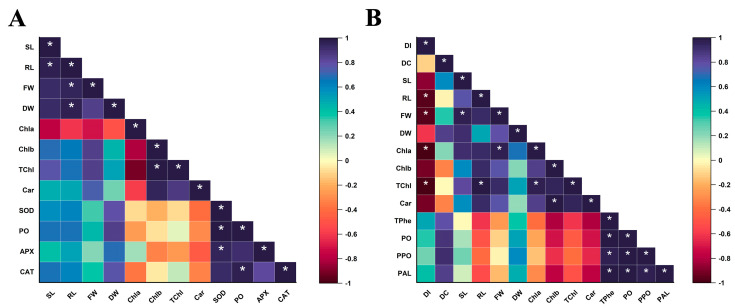
Pearson correlation analysis representing various growth and physiology parameters of tomato plants grown under salinity (**A**) and *Fusarium* wilt (**B**) stress along with *B. subtilis* and *B. amyloliquificiens* inoculation. Abbreviations are displayed as shoot length (SL), root length (RL), fresh weight (FW), dry weight (DW), chlorophyll a (Chla), chlorophyll b (Chlb), total chlorophyll (TChl), carotenoid (Car), disease index (DI), disease control (DC), peroxidase (PO), sodium oxide dismutase (SOD), ascorbate peroxidase (APX), catalase (CAT), polyphenol oxidase (PPO), phenylalanine ammonia-lyase (PAL), and total phenolics (TPhe). *p* = 0.05 (*).

The correlation analysis showed that significant correlations existed between plant growth parameters and physiological attributes under both salinity and biotic stress conditions (Figure 7A,B). Some differences were also observed between the correlation analysis of abiotic and biotic stress scenarios. Here, regardless of stress type, plant growth attributes and antioxidant and phenylpropanoid enzyme activities displayed close positive correlation with each other. A similar effect was seen for total chlorophyll content. For *Fusarium* wilt stress, disease control (DC) was positively correlated with the total phenolic contents (TPhe) and PO, PPO, and PAL activities. 

## 4. Discussion

Plants must survive under different stresses during the whole life cycle [35]. The application of agrochemicals, including pesticides and fertilizers, seems necessary for agricultural production to meet food demands and to prevent losses from biotic and abiotic stresses. The unwise use of agrochemicals has caused the loss of soil biodiversity and raised concerns about human health and safety [36,37]. An alternative to agricultural sustainability is using beneficial microorganisms found in the rhizosphere of plants to protect plants from biotic and abiotic stresses. *Bacillus* genera are considered well-adapted to rhizospheric soil [38]. According to several studies, *Bacillus* species have demonstrated effectiveness as biological control agents, promoters of plant growth, and stimulators of tolerance to various abiotic stresses [39]. Here, we assessed how two *Bacillus* strains (*B. subtilis* strain IS1 and *B. amyloliquificiens* strain IS6) affect tomato plants to ameliorate the growth of tomato plants under salinity and *Fusarium* wilt disease stress. 

Applying some *Bacillus* strains is an effective tool to increase plant growth. In preliminary experiments, both bacterial strains increased the growth of tomato plants when applied using the soil drench method. A significant increase in plant shoot length and dry biomass accumulation was seen. Nonetheless, *Bacillus* strains can positively affect the growth parameters by producing indole acetic acid, cytokinins, and gibberellins and enhanced nutrient uptake by the plants from the surrounding soil [40]. Comparatively, *the B. subtilis* strain IS1 was more responsive than the *B. amyloliquificiens* strain IS6. Such variability has been previously reported in plant growth promotion experiments. Akinrinlola et al. [41] reported varied responses of *Bacillus* strains to promote plant growth during pot trials performed on soybean and wheat plants. This could be due to the variations in *Bacillus* strains regarding mechanisms primarily involved in plant growth promotion. 

Next, tomato plants were exposed to different salinity levels to adopt other salinity levels, ensuring maximum damage while retaining plant viability for further pot trials. Similarly, the pathogenicity of *F. oxysporum* conformed with the tomato variety “Roma” that was used in pot trials. Based on the preliminary trials, the salinity level of 150 mM NaCl was selected, and *F. oxysporum* was found to be pathogenic against the tomato variety used in this experiment. Afterward, tomato plants were raised in a potting mix drenched with both bacterial strains and subsequently challenged with the salinity stress and *Fusarium* wilt pathogen separately. 

We analyzed plant growth traits and found that salt stress significantly reduced plant growth. Salt stress impairs osmosis, imbalances plant nutrition, and, consequentially, lowers plant growth and photosynthesis [42]. In contrast to the untreated plants, *B. subtilis* strain IS1 significantly increased plant height, root length, and biomass accumulation under both stresses. Here, *B. subtilis* strain IS1 enhanced tomato plants’ shoot length, root length, and biomass accumulation by not less than 45% in comparison to salinized control plants. Chlorophyll absorbs light energy and regulates the plant’s growth and development. These pigments also play a role in response to environmental stressors [43]. Carotenoids participate in cell signaling and the production of abscisic acid, which regulates plant growth [44]. Similarly, B. subtilis strain IS1 in tomato plants raised under salinity-stress-enhanced chlorophyll and carotenoid contents. The application of the *B. subtilis* strain IS1 proved that it could restore normal physiological characteristics more effectively, followed by *B. amyloliquificiens* strain IS6 compared to control plants under salt stress. Similar findings were reported by Kumar et al. [45]. They described the restoration of rice plant growth and physiological functions through *Bacillus pumilus* strain JPVS11 inoculation under salinity stress. In another study performed by Ayaz et al. [46], *Bacillus* strains NMCN1 and LLCG23 effectively increased the growth of wheat tolerance towards salt stress. 

Additionally, *B. subtilis* IS1 was the most successful strain in decreasing the severity of *Fusarium* wilt disease (Figure 3). It also significantly promoted the plant growth traits compared with the pathogen control (Table 2). Different previous studies have demonstrated the effectiveness of *Bacillus* species in managing *Fusarium* wilt disease. According to Ramírez et al. [47], *Bacillus cereus* strain MH778713 protected tomato plants against *Fusarium* wilt disease and promoted plant growth. According to Abdallah et al. [48], *Bacillus subtilis* strain SV41 and *B. amyloliquefaciens* subsp. *plantarum* SV65 reduced the severity of tomato *Fusarium* wilt and increased the expression of the lipoxygenase, acidic PR-1, and PR-3 genes. Bacterial biocontrol agents comprise multiple phytobeneficial traits, including the production of antimicrobial compounds, which allow them to suppress the population of plant pathogens in the rhizosphere [21,22]. *Bacillus* strains can secrete extracellular enzymes that might cause the lysis of fungal mycelium [20]. Conversely, the bacterial biocontrol agents can also produce siderophores and phytohormones along with the antimicrobial substances, which might be linked with the increased growth of tomato plants [20]. This study agrees with the previous studies that some *Bacillus* strains, including *B. subtilis*, suppressed *Fusarium* wilt disease and enhanced the growth of potato [49] and cucumber plants [50]. 

Plants have an antioxidant defense system and phenylpropanoid pathway to cope with biotic and abiotic stress [51]. Enzymes involved in the antioxidant defense system (PO, SOD, CAT, and APX) and phenylpropanoid pathway (PO, PPO, and PAL), along with the phenolic compounds, are essential biomarkers to analyze the state of defense against stress conditions [52]. Plants inoculated with beneficial microbes could increase the production of mentioned enzymes and phenolic contents, enabling them to withstand biotic and abiotic stress [53]. Under salinity stress, the application of *B*. *subtilis* IS6 increased the activities of antioxidant-related enzymes (POD, SOD, CAT, and APX) in tomato plants. The onset of salinity stress increases the production of reactive oxygen species (ROS). Their over-accumulation generates oxidative stress and negatively affects plant growth. Peroxidase (POD) enzymes catalyze the oxidation of hydrogen peroxide [54]. Similarly, superoxide dismutase (SOD) provides primary defense against abiotic stress conditions via the dismutation of superoxide anion into hydrogen peroxide and oxygen [55]. Ascorbate peroxidase (APX) catalyzes the conversion of H_2_O_2_ into water [56]. Previous studies have also shown that inoculating plants with *Bacillus* strain increases activities of these key antioxidant enzymes under saline conditions in rice [57] and maize [58], thus improving plants’ growth and biomass. 

In the case of *Fusarium* wilt, tomato plants treated with *Bacillus* strains (*B. subtilis* strain IS1 and *B. amyloliquificiens* strain IS6) showed increased accumulation of total phenolic compound, and activities of POD, POD, and PAL enzymes. *Bacillus* species have been shown to significantly increase the activities of enzymes involved in the phenylpropanoid pathway in different crop plants. Babu et al. [59] found that PGPR substantially boosted the activities of polyphenol oxidase and peroxidase in tomato plants against early blight disease. *B. subtilis* strain GBO3 and *B. pumilus* strain SE34 increased the production of POD, PPO, and PAL in rice plants against leaf blight disease [60]. Phenolic compounds act as chemical defense barriers against fungal plant pathogens and are found in increased quantities in disease-resistant plant varieties [61]. POD oxidatively polymerizes hydroxycinnamyl alcohols to produce lignin in plant cell walls. PAL catalyzes the formation of cinnamic acid [62]. PPO converts the phenolic compounds to free radicals, thus creating an unfavorable toxic environment for pathogens [63]. The increased accumulation of phenolic compounds and defense-related enzymes (PPO, POD, and PAL) must play a key role in defending against *Fusarium* wilt disease. 

## 5. Conclusions

The findings presented in this study highlight the significance of *B. subtilis* strain IS1 and *B. amyloliquificiens* strain IS6 in protecting tomato plants against multiple stresses. In conclusion, the use of *B. subtilis* strain IS1 in tomato plants as soil drench prevented the adverse effects of salinity stress and *Fusarium* wilt disease and enhanced plant growth through induced stress tolerance. 

## Figures and Tables

**Table 1:** Details of the treatment.

No.	Treatment Description
1	Non-treated control (UC)
2	Plants receiving NaCl (150 mM) solution alone as salinity control (SS)
3	Plants receiving *F. oxysporum* spore suspension alone as pathogen control (Fol)
4	Plants receiving NaCl solution and IS1 (SS + IS1)
5	Plants receiving NaCl solution and IS6 (SS + IS6)
6	Plants receiving *F. oxysporum* spore suspension and IS1 (Fol + IS1)
7	Plants receiving *F. oxysporum* spore suspension and IS6 (Fol + IS6)

**Table 2:** Effect of *B. subtilis* IS1 and *B. amyloliquificiens* IS6 inoculation on growth parameters of tomato plants under salinity and *Fusarium* wilt disease stress.

Treatments		Shoot Length (cm)	Root Length (cm)	Fresh Biomass (g)	Dry Biomass (g)
Non-treated Control		17.83 ± 1.28 ^b^	08.96 ± 0.34 ^b^	25.36 ± 1.17 ^b^	01.96 ± 0.07 ^cd^
Salinity Stress	SS	14.42 ± 0.95 ^c^	06.14 ± 0.26 ^cd^	18.27 ± 1.32 ^de^	01.65 ± 0.09 ^de^
	IS1	20.84 ± 1.36 ^a^	12.39 ± 1.03 ^a^	29.63 ± 2.06 ^a^	02.47 ± 0.13 ^a^
	IS6	17.87 ± 1.71 ^b^	08.05 ± 0.59 ^b^	22.18 ± 1.38 ^b–d^	02.03 ± 0.16 ^c^
*Fusarium* Wilt	*F. oxysporum*	12.03 ± 0.86 ^cd^	05.27 ± 0.38 ^c–e^	14.04 ± 1.62 ^f^	01.32 ± 0.08 ^f^
	IS1	18.96 ± 1.82 ^b^	07.10 ± 0.57 ^bc^	24.12 ± 1.88 ^bc^	02.16 ± 0.17 ^b^
	IS6	15.35 ± 1.19 ^c^	06.83 ± 0.29 ^b–d^	21.06 ± 1.31 ^cd^	02.04 ± 0.16 ^c^

Values are presented as mean ± standard error. A similar small letter depicts non-significant differences.

**Table 3:** Effect of *B. subtilis* IS1 and *B. amyloliquificiens* IS6 inoculation on chlorophyll and carotenoid contents of tomato plants under salinity and *Fusarium* wilt disease stress.

Treatments		Chl a (mg g^−1^)	Chl b (mg g^−1^)	Total Chl (mg g^−1^)	Carotenoid (mg g^−1^)
Non-treated Control		1.07 ± 0.062 ^ab^	0.62 ± 0.041 ^a^	1.65 ± 0.527 ^a^	0.38 ± 0.044 ^a^
Salinity Stress	SS	0.81 ± 0.075 ^cd^	0.43 ± 0.037 ^cd^	1.27 ± 0.096 ^de^	0.26 ± 0.025 ^cd^
	IS1	1.13 ± 0.071 ^a^	0.56 ± 0.078 ^b^	1.61 ± 0.124 ^ab^	0.32 ± 0.027 ^b^
	IS6	1.01 ± 0.108 ^bc^	0.51 ± 0.031 ^bc^	1.56 ± 0.116 ^bc^	0.28 ± 0.024 ^c^
*Fusarium* Wilt	*F. oxysporum*	0.62 ± 0.042 ^d–f^	0.29 ± 0.021 ^g^	0.86 ± 0.076 ^fg^	0.21 ± 0.019 ^ef^
	IS1	0.96 ± 0.097 ^bc^	0.37 ± 0.028 ^c–e^	1.31 ± 0.134^cd^	0.25 ± 0.023 ^cd^
	IS6	0.75 ± 0.087 ^de^	0.33 ± 0.019 ^fe^	1.02 ± 0.095 ^d–f^	0.23 ± 0.021 ^de^

Values are presented as mean ± standard error. Similar small letters depict non-significant differences.

## Data Availability

The original contributions presented in the study are included in the article/Supplementary Materials, further inquiries can be directed to the corresponding authors.

## Supplementary Materials

The following supporting information can be downloaded at: https://www.mdpi.com/article/10.3390/microorganisms12102092/s1, Table S1: Physiochemical properties of soil.
