# Supplementary material for: Exploring the Potential of Bacillus subtilis IS1 and B. amyloliquificiens IS6 to Manage Salinity Stress and Fusarium Wilt Disease in Tomato Plants by Induced Physiological Responses
Source: Microorganisms. 2024 Oct 19;12(10):2092. doi: 10.3390/microorganisms12102092 (PMC11510684; doi:10.3390/microorganisms12102092)
Supplement: Supplementary file 1 [file microorganisms-12-02092-s001.zip › microorganisms-3243086-supplementary.pdf]

Table S1: Physiochemical properties of soil.

Properties	Values
Sand	42.06
Silt	51.13
Clay	6.82
Organic matter (%age)	0.67
Total Nitrogen (%age)	0.073
Available P (mg Kg^{-1})	5.43
Available K (mg Kg^{-1})	121.9
pH	7.9
